# Electroacupuncture at ST36 Protects ICC Networks via mSCF/Kit-ETV1 Signaling in the Stomach of Diabetic Mice

**DOI:** 10.1155/2017/3980870

**Published:** 2017-01-22

**Authors:** Lugao Tian, Beibei Zhu, Shi Liu

**Affiliations:** Division of Gastroenterology, Union Hospital, Tongji Medical College, Huazhong University of Science and Technology, Wuhan, China

## Abstract

*Background*. Electroacupuncture (EA) at ST36 has been used to regulate gastric motility and effectively improve gastric emptying in diabetic patients. Nevertheless, the specific mechanisms underlying the efficacy of this treatment remain unknown. The aim of this study was to assess the variations of interstitial cells of Cajal (ICC) and explore the changes in mSCF/KIT-ETV1 signaling in the antrum and corpus of diabetic mice after treatment with EA.* Methods*. Male C57BL/6 mice were randomized into five groups: control group, diabetic group (DM), diabetic-plus-sham EA group (SEA), diabetic-plus-low-frequency EA group (LEA), and diabetic-plus-high-frequency EA group (HEA). The expression levels of Ano1, c-Kit, and ETV1 were assessed by immunofluorescence in the antrum and corpus. Western blotting and PCR methods were further used to evaluate c-Kit, mSCF, and ETV1 expression.* Results*. (1) c-Kit and Ano1 were obviously decreased in the DM group, but c-Kit reduced much more than Ano1. (2) The mSCF, c-Kit, and ETV1 mRNA and protein levels were obviously decreased in the DM group in both the antrum and the corpus (*P* < 0.01), but they were significantly elevated in the LEA and HEA groups (*P* < 0.01).* Conclusions*. Ano1 is a reliable marker to detect ICC changes in diabetes; low- and high-frequency EA at acupoint ST36 can protect the networks of ICC possibly via normal activation of mSCF/KIT-ETV1 signaling.

## 1. Introduction

The incidence rate of diabetes has been rapidly increasing following the improvements in the standard of living in recent decades. The gastrointestinal (GI) complications of diabetes are primary causes of the lower quality of life in diabetic patients, as well as increased hospitalization and mortality rates. Gastroparesis, defined as delayed gastric emptying without mechanical obstruction, is the most common complication in the stomach due to long-term chronic hyperglycaemia. A series of studies about the mechanisms underlying gastroparesis have been proposed, and different types of injured cells in the stomach may contribute to gastroparesis [1, 2].

Injury of the interstitial cells of Cajal may be one of the most significant factors causing gastrointestinal dysfunction in diabetic patients. Normal gastric emptying depends on the coordination of the contractile activity of the corpus and antrum. Specifically destroying ICC in the antrum and corpus has revealed slow wave dysrhythmia and delayed gastric emptying [3, 4]. Gastroparesis was reported to occur in both humans and animal models with a reduced number of ICC, regardless of whether it was in the region of the myenteric plexus (MY) or intramuscular (IM) [5]. C-Kit is the classical marker of the ICC and has been used to follow the loss of the ICC and variations in the network density associated with various GI disorders. Recently, Ano1, which was first found in gastrointestinal stromal tumour (GIST) and functions as a calcium-activated chloride channel, has been identified as a novel ICC marker in gastrointestine [6]. As a c-Kit-independent marker, Ano1 can label all classes of the ICC in the muscularis propria, even c-Kit dim cells [7]. However, Ano1 immunoreactivity has not been observed in diabetes associated with loss of c-Kit positive ICC.

A few studies have demonstrated that SCF/Kit signaling was necessary for sustaining the ICC phenotypes, proliferation, and differentiation [8]. ICC loss was accompanied with reduced c-Kit and SCF protein in the stomach of diabetic animal models and patients. Recently, ETV1, as the main downstream effector of SCF/Kit signaling, was also found to be necessary for the normal development of ICC. ETV1 is specifically expressed on the ICC-IM and ICC-MY in the stomach, small intestine, and colon and ETV1^−/−^ mice have been shown to have losses of the intramuscular ICC and myenteric ICC [9]. So far, there have been limited reports about the SCF/Kit-ETV1 signaling in diabetes.

Although pharmaceutical intervention and some nondrug treatments have been utilized, the curative effects of diabetic gastroparesis are still unsatisfactory. Electroacupuncture (EA) is currently used as an optimized type of acupuncture and has revealed a potential role in regulating gastric motility and treating gastroparesis [10, 11]. In diabetic animal models, EA has been reported to promote gastric motility and improve delayed gastric emptying [12]. For diabetic patients with gastroparesis, EA could alleviate dyspeptic symptoms and accelerate solid gastric emptying [11]. Although the mechanisms underlying the therapeutic effects of EA have not been systematically investigated, our previous studies have demonstrated that EA at ST36 could rescue the networks of ICC in the stomachs of diabetic rats, which might contribute to the improved gastric emptying [13]. However, it is still unknown whether SCF/Kit-ETV1 signaling participates in the process of EA on protected ICC.

The purposes of this study were to (1) employ c-Kit combined Ano1 to evaluate the changes of ICC in diabetic mice; (2) investigate whether the mSCF/Kit-ETV1 signaling is involved in the protection of the ICC networks in the antrum and corpus of diabetic mice after electroacupuncture at ST36.

## 2. Materials and Methods

### 2.1. Animals

A total of 60 male six- to eight-week-old C57BL/6 mice were used in this study. All of the mice were obtained from Beijing Hua Fu Kang Bio-technology Co., LTD, Beijing, China. They were housed in a standardized laboratory room (22°C, 12/12 h light-dark cycle) and were given food and sterile water ad libitum. Before entering the formal study, the mice had adapted to the laboratory environment for two weeks. The mice received humane care and all of the experiments were approved by the Animal Care and Use Committee of Tongji Medical College, Huazhong University of Science and Technology.

### 2.2. Diabetic Models

After overnight fasting without water-deprivation, diabetes was successfully induced in mice by a single intraperitoneal injection of streptozotocin (STZ, Sigma, St Louis, MO, USA) at a dose of 150 mg/kg that was prepared in 0.1 mol/L citrate buffer (pH 4.5; Sigma, St Louis, MO, USA). The control group was injected with the same volume of citrate buffer. One week after the injection, diabetes was confirmed by measuring the blood glucose level using a drop of whole blood obtained from a small skin incision made at the tip of the tail. Animals were considered diabetic if their blood glucose level was above 250 mg/dL.

### 2.3. Experimental Protocols

All of the mice were randomly divided into five groups (12 mice/group): the control group, the diabetic group (DM), a group of diabetic mice with sham EA (SEA, only acupuncture without electric current), a group of diabetic mice treated with low-frequency EA group (LEA, 10 Hz, 1–3 mA), and a group of diabetic mice treated with high-frequency EA group (HEA, 100 Hz, 1–3 mA). EA was carried out continuously at ST36 for eight weeks. Eight weeks later, all of the mice were sacrificed and specimens of the antrum and corpus were carefully obtained. Part of fresh tissues were used for immunofluorescence. Other part of tissues were stored at −80°C for western blotting and RT-PCR analysis.

### 2.4. Electroacupuncture

Electroacupuncture was performed at 9:00–9:30 AM every day. An electrical stimulator (G6805-2A; Shanghai Huayi Medical Instrument Factory, Shanghai, China) was used to stimulate. The acupoint of ST36 for the mice was located at the posterolateral knee of bilateral hind limbs, approximately 2 mm below the fibular head [14]. For the real EA groups, the bilateral acupoints of hind limbs were inserted with a pair of stainless steel needles (0.16 × 7 mm) at a depth of 2-3 mm. The electrical current was satisfied until the bilateral hind limbs started to tremble slightly and sustained as long as 30 minutes. The sham group was performed as the real EA groups only without electric current. To eliminate the influence of stress, animals were restrained in a cage for 30 min/d at 9:00–9:30 AM before EA for two weeks.

### 2.5. Immunofluorescence Staining

Tissues of four or five mice for each group were used to perform immunofluorescence staining. The freshly obtained mouse stomachs were placed in Kreb's solution (mmol/L): 118.1 NaCl, 4.8 KCl, 25 NaHCO_3_, 1.0 NaH_2_PO_4_, 1.2 MgSO_4_, 11.1 Glucose, 2.5 CaCl_2_, and pH 7.3–7.4 after being bubbled with 95% O_2_ and 5% CO_2_. The stomach was opened along the lesser curvature and the gastric contents were washed away. The tissue was then pinned in a dish that was coated with Sylgard with the mucosa face up. The mucosa was carefully peeled away with forceps. The antrum and corpus whole-mount preparations were fixed with ice-cold acetone for ten minutes. Following fixation, the preparations were rinsed three times (ten minutes each time) in 1x PBS. Nonspecific binding was blocked in PBS containing 5% normal goat serum and 0.5% Triton X-100. The tissues were next incubated for 48 hours at 4°C with the primary antibody diluted in primary antibody dilution buffer containing 0.3% Triton X-100. There were two pairs of primary antibodies used: rat anti-c-Kit (clone: ACK2; 1 : 100; eBioscience, San Diego, CA, USA) with rabbit anti-Ano1 (1 : 200, Abcam, Cambridge, MA, USA), rat anti-c-Kit (clone: ACK2; 1 : 100; eBioscience, San Diego, CA, USA), and rabbit anti-ETV1 (1 : 200, Abcam, Cambridge, MA, USA) that were used for incubations. After washing with PBS, the specific labelling was evaluated by incubation with Dylight 488 with goat anti-rat IgG (Abbkine) and Dylight 594 with goat anti-rabbit IgG (Abbkine) diluted in PBS containing 0.5% Triton X-100 for two hours. The incubation solution without primary antibodies was regarded as the negative control. A confocal microscope (Olympus, Tokyo, Japan) was used to examine the preparations.

### 2.6. Western Blots Analysis

Tissues of five mice for each group were applied to western blots analysis. Fresh-frozen antrum and corpus specimens were homogenized separately in RIPA buffer with protease inhibitor. Then, the homogenates were centrifuged at 12000 rpm for 15 min at 4°C and all of the supernatants were collected as the protein. The concentration of the protein was measured by the bicinchoninic acid (BCA) method. Next, equivalents of 80 mg of extracted proteins were separated by 10% sodium dodecyl sulphate-polyacrylamide gel electrophoresis (SDS-PAGE), and the separated proteins were transferred to PVDF membranes. The nonspecific binding sites were subsequently blocked in 5% nonfat dry milk dissolved in Tris-buffered saline containing 0.1% Tween 20 (TBST). One hour later, all of the membranes were incubated with primary antibodies polyclonal goat anti-c-Kit (1 : 1000; R&D Systems, Minneapolis, USA), goat anti-SCF (1 : 1000; R&D Systems, Minneapolis, USA), or rabbit anti-ETV1 (1 : 500; Abcam, Cambridge, MA, USA) overnight at 4°C. Rabbit anti-mouse GAPDH (1 : 2000; GeneTex, San Antonio, TX, USA) served as the internal control. After being washed three times in TBST, the membranes were incubated with a HRP-linked secondary antibody (HRP-linked goat anti-rat or HRP-linked goat anti-rabbit, 1 : 2000) for one hour at room temperature. After being washed three times in TBST, detection of the bands was performed by a chemical reaction with the enhanced chemiluminescence reagents (ECL; ThermoFisher, USA), and the blot was subjected to autoradiography. The Quantity One software (Bio-Rad Technical Service Department, Version 4.6.2) was applied to measure the band intensity.

### 2.7. RNA Extraction and Quantitative Real-Time PCR Analysis

Tissues of six mice were used to implement quantitative real-time PCR analysis. The total RNA of the antrum and corpus tissues from mice were extracted with the TRIzol reagent (Invitrogen, Carlsbad, CA, USA). Then, the RNA was converted into cDNA using PrimeScript RT Master Mix (Takara, Otsu, Japan). Real-time quantitative reverse-transcriptase PCR (qRT-PCR) was performed using SYBR Premix Ex TaqII (Takara) on a Roche LightCycler^R^480 (Roche, Switzerland). The specific primer sequences used were as follows: c-Kit mRNA-5′-GACCCGACGCAACTTCCTTA-3′ and 5′-GAGCATCTTCACGGCAACTGT-3′, mSCF mRNA-5′-GGAAAATAGTGGATGACCTCGTG-3′ and 5′-TGGAATCTTTCTCGGGACCTAAT-3′, ETV1 mRNA-5′-GTCCATACCAGACAGCACCTACC-3′ and 5′-GAA GGGGATGTTTGGCTCAG-3′, GAPDH mRNA-5′-TTCACCACCATGGAGAAGGC-3′, and 5′-GGCATGGACTGTGGTCATGA-3′. The transcript level was normalized by the glyceraldehyde-3-phosphate dehydrogenase (GAPDH) and the relative expression level of each target gene was calculated with the power formula: 2^−ΔCT^ (ΔCT = CT_Target_−CT_GAPDH_).

### 2.8. Statistical Analysis

All values were expressed as the means ± SEM and a one-way ANOVA was applied to compare the differences among multiple groups. Pearson's correlation and linear regressions were used to evaluate the relationships between the mSCF and ETV1 protein expression. A value of *P* < 0.05 was regarded to be statistically significant. The statistical analysis was performed using with SPSS 17.0 (SPSS Inc., Chicago, IL).

## 3. Results

### 3.1. Effects of EA on the Expression of c-Kit and Ano1 in the ICC

**Figure 1:**
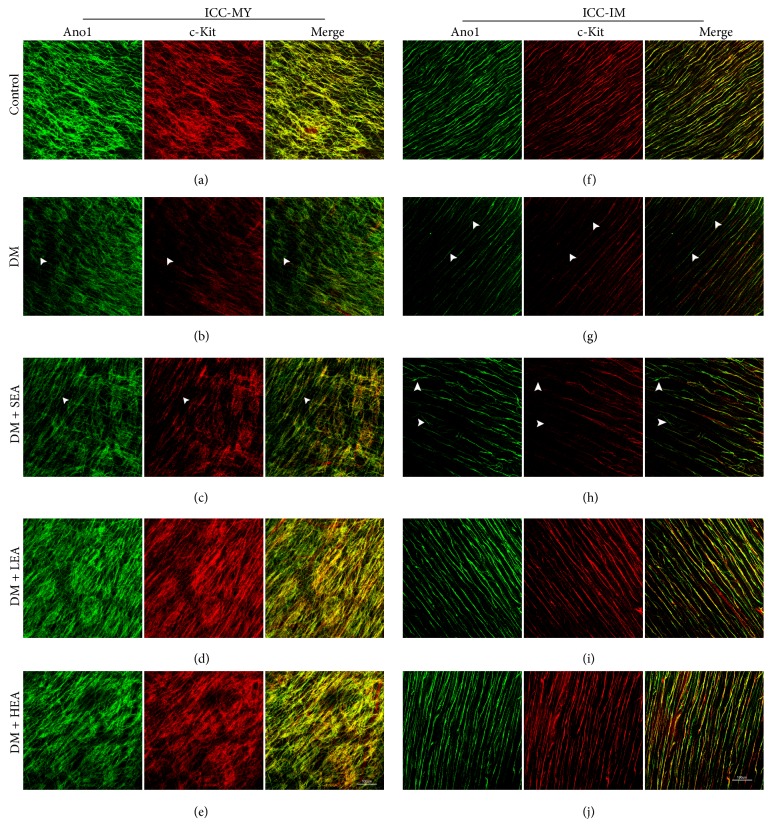
Immunofluorescence of the ICC-MY (a)–(e) and ICC-IM (f)–(j) in the antrum labelled with Ano1 (green) and c-Kit (red). In the control group, the ICC networks of Ano1 and c-Kit were nearly completely overlapping, and both of the labels showed dense and intact ICC networks. The networks of the ICC in the MY and IM were incomplete, with destroyed processes in the DM and SEA groups. However, in the LEA and HEA groups, the ICC networks had nearly recovered to the normal level, and the two markers of the ICC indicated that there were long and abundant branches. The white arrowheads showed Ano1 positive c-Kit dim or negative cells. Scale bars = 100 *μ*m and refer to all panels.

**Figure 2:**
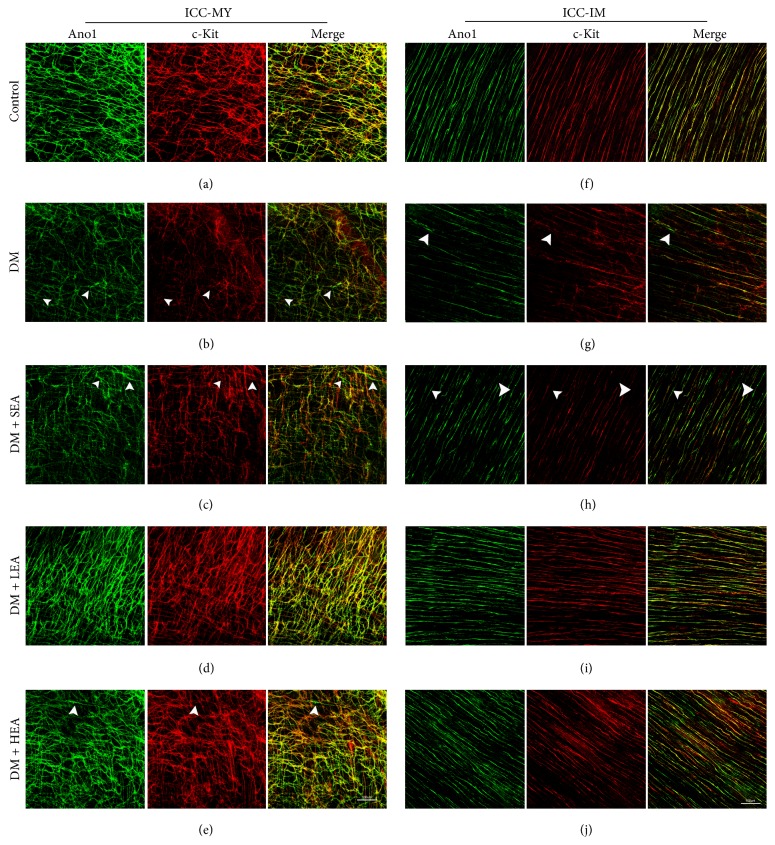
Immunofluorescence of the ICC-MY (a)–(e) and ICC-IM (f)–(j) in the corpus labelled with Ano1 (green) and c-Kit (red). In the control group, the ICC networks indicated by Ano1 and c-Kit were all bright, and the integrity was maintained in both MY and IM. The two markers for the ICC networks were very dim, and the networks were severely disrupted in the DM group. Similar changes in the ICC networks were also observed in the SEA group. However, in the LEA and HEA groups, ICC-MY and ICC-IM still remained relatively intact, and the brightness of the two markers was almost normal. The white arrowheads show the Ano1 positive c-Kit dim or negative cells. Scale bars = 100 *μ*m and refer to all panels.

Double labelling of c-Kit and Ano1 (Figures 1 and 2) was performed to evaluate the changes in the ICC networks in the antrum and corpus. In the control group, Ano1- or c-Kit-positive cells were connected to each other and formed intact cellular networks. The two markers revealed near 100% colocalization on ICC-MY and ICC-IM in both the antrum and the corpus (Figures 1(a), 1(f), 2(a), and 2(f)). However, in the DM group, there was reduced Ano1, which almost paralleled the reduction in c-Kit for the ICC-IM and ICC-MY in the antrum and corpus (Figures 1(b), 1(g), 2(b), and 2(g)). The networks of the ICC for the two markers became very sparse and the processes were heavily disrupted. However, the c-Kit level was reduced much more obviously than the Ano1 and a small number of Ano1- positive c-Kit-dim cells were found in the ICC-MY and ICC-IM (white arrowheads). Similar findings were noted in the sham group (Figures 1(c), 1(h), 2(c), and 2(h)). Nevertheless, no significant changes were observed in the LEA and HEA groups, and the morphology and density of Ano1 and c-Kit for the ICC remained near the normal levels in the antrum and corpus (Figures 1(d), 1(e), 1(i), 1(j), 2(d), 2(e), 2(i), and 2(j)).

**Figure 3:**
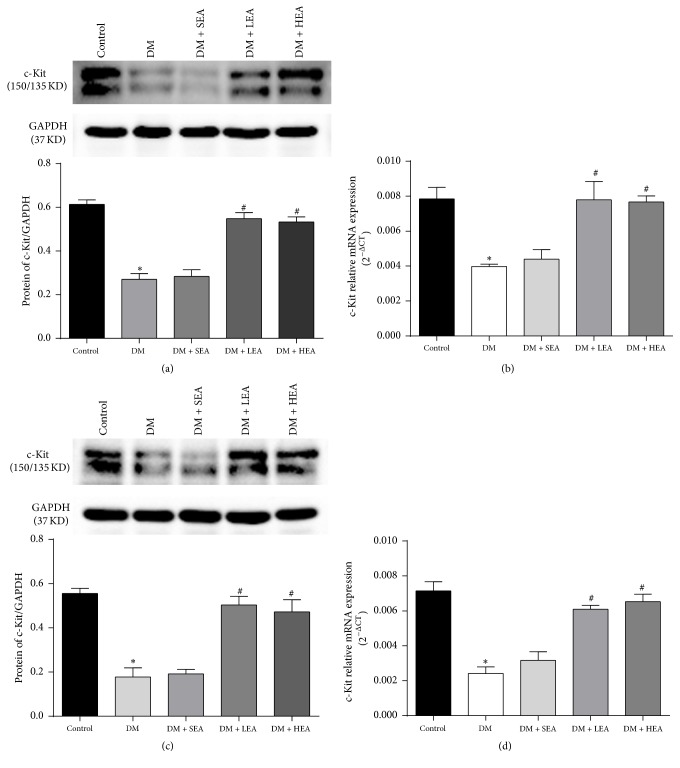
Expression of c-Kit protein and mRNA in the antrum (a,b) and corpus (c,d). Compared with the control group, the expression of the c-Kit protein in the antrum and corpus was obviously decreased in the DM group. In contrast, it was significantly increased in the LEA and HEA groups compared to the DM group. The same situation was noted for the mRNA expression in the antrum and corpus. ^*∗*^*P* < 0.05 compared with the control group and ^#^*P* < 0.05 compared with the DM group.

To further assess the changes in c-Kit expression, its expression was detected at both the protein and mRNA levels in the antrum and corpus (Figure 3). Compared to the control group, the protein expression of c-Kit in the DM group was obviously decreased in both the antrum (*P* < 0.01) and the corpus (*P* < 0.01) (Figures 3(a) and 3(c)). However, significant increases were found in both the LEA group and the HEA group compared with the DM group in the antrum (*P* = 0.000; *P* < 0.01) and corpus (both *P* < 0.01). However, there were no significant differences in the protein expression of c-Kit between the SEA group and the DM group in either the antrum (*P* = 0.718) or the corpus (*P* = 0.797). The mRNA expression showed a tendency similar to the protein expression in the antrum and corpus (Figures 3(b) and 3(d)).

### 3.2. Effects of EA on mSCF Expression

**Figure 4:**
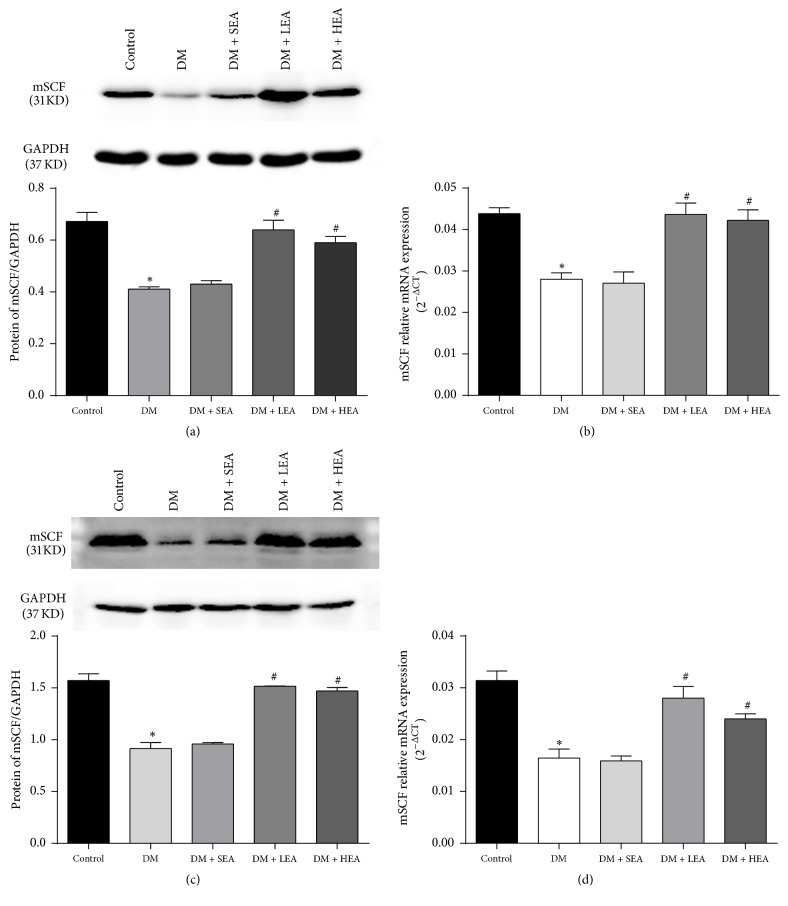
Expression of the mSCF protein and mRNA in the antrum (a,b) and corpus (c,d). In the antrum and corpus tissues, the mSCF protein level was obviously decreased in the DM and SEA groups compared with the control group. However, the expression of the ETV1 protein recovered to near the normal level in the LEA and HEA groups. No significant difference was found between the DM group and the SEA group. The mRNA expression of mSCF showed the same trends as the protein expression in each group. ^*∗*^*P* < 0.05 compared with the control group and ^#^*P* < 0.05 compared with the DM group.

Compared with the control group, the mSCF protein expression (Figures 4(a) and 4(c)) was decreased in the antrum (*P* < 0.01) and corpus (*P* < 0.01) in the DM group. However, compared to the DM group, the expression of mSCF protein was dramatically elevated in both the LEA and the HEA groups in both the antrum (both *P* < 0.01) and corpus (*P* = 0.000, *P* < 0.01). There were also no significant differences between the SEA group and the DM group in terms of the expression level of mSCF protein in either the antrum (*P* = 0.603) or corpus (*P* = 0.478). An analogous situation was found for the mRNA expression (Figures 4(b) and 4(d)).

### 3.3. Effects of EA on ETV1 Expression

**Figure 5:**
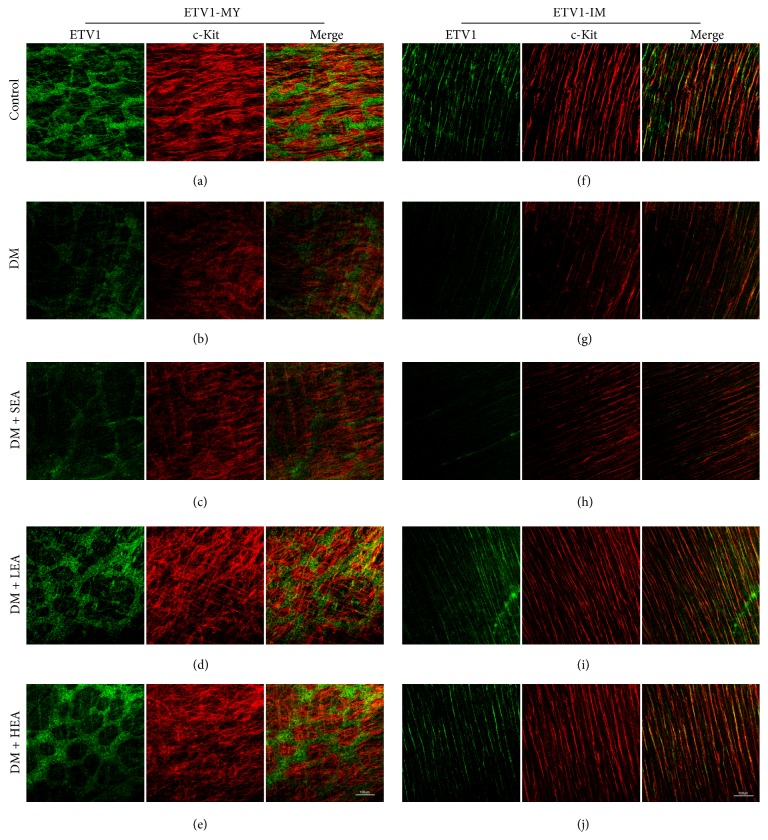
Immunofluorescence of ETV1-MY (a)–(e) and ETV1-IM (f)–(j) in the antrum labelled with ETV1 (green) and c-Kit (red). In the control group, the networks of ETV1 were very bright and abundant in the MY and closely followed the networks of c-Kit. However, the expression of ETV1 was relatively decreased in the IM and the morphology was similar to that in the ICC. The ETV1-IM overlapped with c-Kit or close to the networks of the ICC. As the networks of the ICC, ETV1-MY and ETV1-IM were heavily damaged in the DM and SEA groups. However, the brightness and networks of ETV1 had almost recovered to the normal level in the LEA and HEA groups. Scale bars = 100 *μ*m and refer to all panels.

**Figure 6:**
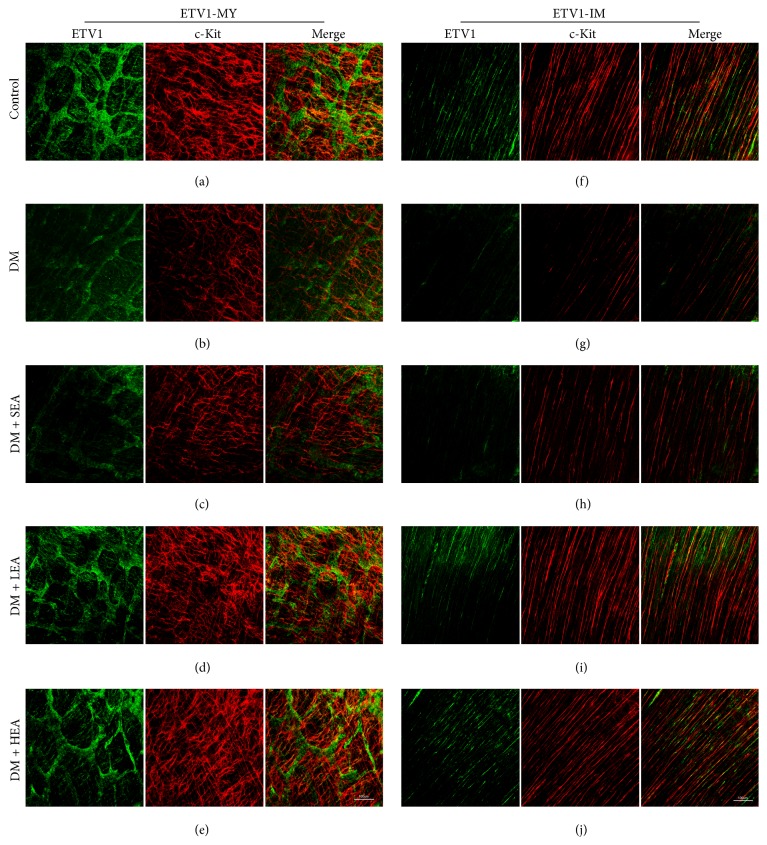
Immunofluorescence of ETV1-MY (a)–(e) and ETV1-IM (f)–(j) in the corpus labelled with ETV1 (green) and c-Kit (red). The expression of ETV1 was abundant in the MY but relatively lower in the IM in the control group. The networks of both ETV1 and c-Kit were bright and intense. The ETV1 networks moved tightly around the c-Kit network and partially overlapped. In the DM group, the networks of ETV1 (as the ICC networks) were severely disrupted and the brightness was obviously reduced. The SEA group showed the same trends as the DM group. However, no obvious changes were found in the LEA and HEA groups compared with the control group. Scale bars = 100 *μ*m and refer to all panels.

ETV1 and c-Kit were costained by immunofluorescence in the antrum and corpus (Figures 5 and 6). In the control group, ETV1 was abundantly expressed in the MY, and the ETV1 distribution was closely associated with the networks of the ICC (Figures 5(a) and 6(a)). In the intramuscular region, the expression of ETV1 became relatively decreased, and the morphology was bipolar similar to that in the ICC (Figures 5(b) and 6(b)). The ETV1 and c-Kit expressions overlapped tightly or were closely associated. The decreased ETV1 in the DM group was paralleled by a decrease in the c-Kit expression (Figures 5(c) and 6(c)). The networks of ETV1 were disrupted similar to those of c-Kit, and the brightness was greatly reduced compared to the control group. In the LEA and HEA groups, the ETV1 expression was obviously restored in the antrum and corpus (Figures 5(d), 5(e), 5(i), 5(j), 6(d), 6(e), 6(i), and 6(j)). Both the density and the brightness of ETV1, as well as networks of c-Kit, appeared to be nearly the same as the normal level.

**Figure 7:**
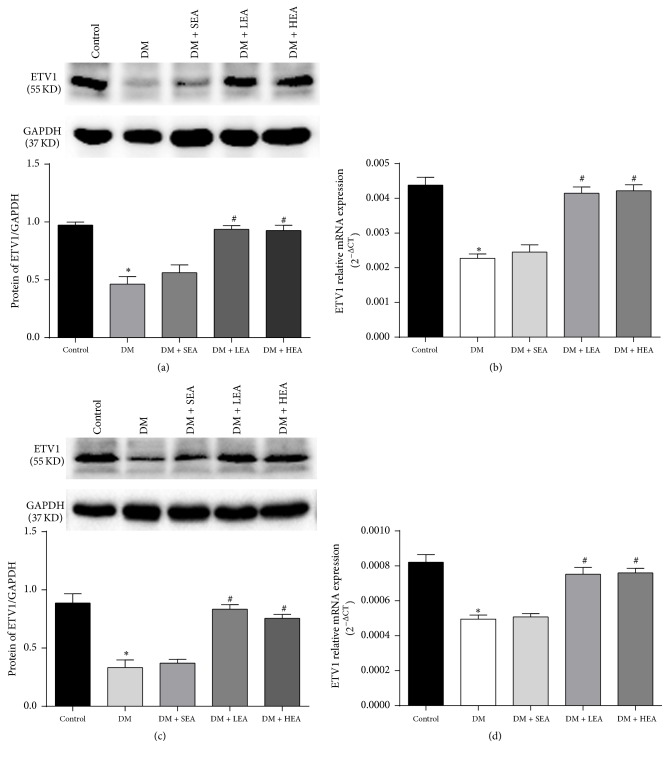
Expression of ETV1 protein and mRNA in the antrum (a,b) and corpus (c,d). Compared to the control group, the ETV1 protein expression was severely reduced in the antrum and corpus tissues in the DM and SEA group. However, no obvious changes were found in the LEA and HEA groups. The mRNA in each group showed the same trends as the ETV1 protein expression in the antrum and corpus. ^*∗*^*P* < 0.05 compared with the control group and ^#^*P* < 0.05 compared with the DM group.

A Western blot analysis was used to further measure the ETV1 protein expression in the antrum and corpus (Figure 7). As observed by immunohistochemistry, the ETV1 protein expression was obviously downregulated in the DM group in the antrum (*P* < 0.01) (Figure 7(a)) and corpus (*P* = 0.000) (Figure 7(c)) compared with the control group. The protein expression levels in the LEA and HEA groups were dramatically increased compared to the DM group in both the antrum (*P* = 0.000; *P* < 0.01) and the corpus (both *P* < 0.01). However, no differences were found in the ETV1 expression levels between the DM group and the SEA group in either the antrum (*P* = 0.182) or the corpus (*P* = 0.624). Similar changes in ETV1 mRNA expression were observed in the antrum and corpus (Figures 7(b) and 7(d)).

### 3.4. Correlation and Regression Analysis

**Figure 8:**
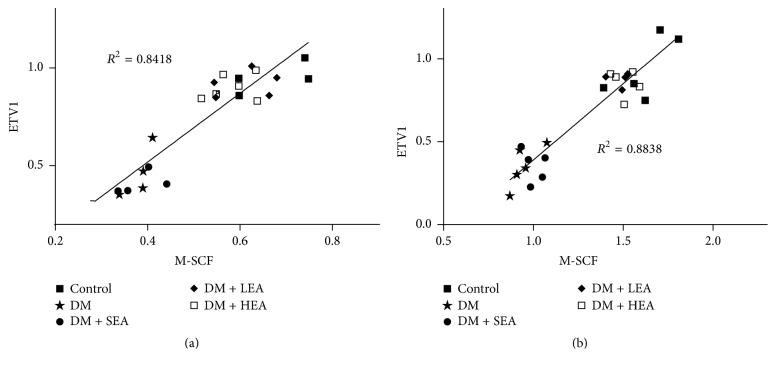
Correlations between the mSCF protein expression and ETV1 protein expression in the antrum (a) and corpus (b). A significant correlation between mSCF and ETV1 was found in the antrum (*R*^2^ = 0.8418, *P* < 0.01) and corpus (*R*^2^ = 0.8838, *P* < 0.01). Each point represents the value from an individual mouse (*N* = 25).

To evaluate the relationship between mSCF and ETV1, Pearson's correlation and linear regression analyses were applied to examine the differences in the expression of the mSCF and ETV1 proteins. Pearson's correlations between mSCF and ETV1 were determined in the antrum and corpus (*r* = 0.917 (*P* = 0.000) and *r* = 0.940 (*P* = 0.000), resp.). In the linear regression analysis, mSCF was entered as a dependent variable for both the antrum (*R*^2^ = 0.8418, *P* < 0.001) (Figure 8(a)) and corpus (*R*^2^ = 0.8838, *P* < 0.001) (Figure 8(b)). The regression equations for the antrum and corpus were *Y* = −0.1832 + 1.7553*X* and *Y* = − 0.5322 + 0.9214*X*, respectively.

## 4. Discussion

In our current study, we have accurately exhibited the changes of the networks of the ICC and the expression of molecules involved in mSCF/Kit-ETV1 signaling in the antrum and corpus of STZ-induced diabetic mice. After the mice were treated with EA, the mSCF/Kit-ETV1 signaling sustained the normal activated state and networks of the ICC to near the normal level.

Acupuncture is a traditional Chinese medicine that has been used for thousands of years. Electroacupuncture, which is a modified form of acupuncture, has recently attracted many researchers' attention due to its safety and cost-effectiveness. On the aspect of regulating gastrointestinal motility, ST36 (Zusanli) is the most commonly used point and different frequencies are employed. For example, several studies have demonstrated that gastric emptying could be accelerated by low-frequency EA at ST36 in animal models [10, 15]. But hardly no studies have reported EA with high frequency to regulate gastric motility and only a few researches found that high-frequency EA could promote esophageal and colonic motility [16, 17]. In our former study, we have shown that both low- (10 Hz) and high- (100 Hz) frequency EA at ST36 could enhance the gastric emptying in diabetic rats [13]. The ICC is the origin of slow waves, and the critical mediators of neurotransmission on regulating gastric motility have been well established. The accelerative effects of EA on gastric emptying may involve the protection of the damaged ICC networks in diabetes. Recently, EA with low frequency have been proposed to rescue ICC in gastrointestine [18]. Our previous research also further verified that, in diabetic rats, both low- (10 Hz) and high- (100 Hz) frequency EA could protect networks of ICC [13]. So, in this study, the chosen frequencies (10 Hz and 100 Hz) were based on prevenient study that indicated stimulatory effect of gastric motility and function of protecting the ICC.

Antibodies against c-Kit have been extensively applied to characterize the ICC changes in diabetes. Recently, a few studies have adopted Ano1 to detect all classes of ICC in the stomachs of humans and mice [6]. Ano1 is a Kit-independent marker of the ICC without sequence homology to the peptide sequence of c-Kit and no cross-reaction with c-Kit on the ICC [19]. Compared with using only c-Kit, some studies have employed Ano1 and c-Kit costaining to detect the ICC and have shown different results, especially under pathological conditions. In the colons of patients with slow transit constipation or W/W^V^ mice, a few Ano1 positive c-Kit dim or negative cells were found [20, 21]. Thus, if only c-Kit had been used to label the ICC, these cells might have been lost. Another advantage of using Ano1 is that c-Kit-expressing mast cells are not Ano1 positive. These findings suggest that Ano1 is more specific and sensitive for detecting ICC networks than c-Kit. To the best of our knowledge, the current study is the first time that, the combination of c-Kit and Ano1 immunohistochemistry was employed to evaluate the changes to the ICC in a mouse model of diabetes and EA. The alterations of Ano1 and c-Kit were consistent with our previous studies that had only used c-Kit as a marker of the ICC in diabetic rats [13]. Perfect colocalization of c-Kit and Ano1 could be found in both the ICC-MY and the ICC-IM of the normal antrum and corpus, but the observed discrepancies in the samples from mice with diabetes and nonspecific staining of c-Kit suggest that Ano1 is a more reliable marker for quantifying the ICC in the stomachs of diabetic mice. Because c-Kit has an important role in the development and maintenance of the ICC, changes in its phenotype may temporally precede changes in the amount of ICC. In response to the hostile environment, ICC transdifferentiation and apoptosis have been suggested [22, 23]. Thus, we speculate the Ano1 positive c-Kit dim cells that were found in this study may be ongoing transdifferentiated or apoptotic ICC. In accordance with our former results, there were no obvious discrepancies found between the low- and high-frequency EA with regard to the density of c-Kit and Ano1 staining. It is possible that eight weeks of EA were not sufficient to distinguish the effects of the two frequencies. The recovered networks of the ICC that were labelled by Ano1 and c-Kit suggested that low- and high-frequency EA could rescue the ICC-MY and ICC-IM in the antrum and corpus of diabetic mice, which might further contribute to the gastric motility.

The stem cell factor (SCF), as the ligand of c-Kit, can activate the c-Kit receptor protein and sustain ICC development. ICC proliferation and differentiation have been proposed to result from the activation of SCF/Kit signaling [8]. Two forms of SCF exist: a transmembrane protein (mSCF) and a soluble protein (sSCF). However, the mSCF may preferentially contribute to the differentiation, survival, and maintenance of the ICC [24]. Our previous study also suggested that the mSCF was a more effective agonist for c-Kit than sSCF [25]. Therefore, in this study, we focused on the mSCF to evaluate alterations in SCF/Kit signaling which may result in ICC changes. The upregulation of mSCF and c-Kit by low- and high-frequency EA suggested that mSCF/Kit signaling might be involved in the protection of the ICC in diabetic mice.

As mentioned above, ETV1 is the major downstream effector of mSCF/Kit signaling. Nevertheless, at present, no reports about ETV1 have been published in models of diabetes and EA. This is because, as an ETS family transcription factor, the focus on ETV1 has been on ETS-dependent tumours, especially for the development of gastrointestinal stromal tumours [9, 26]. In the normal gastric tissues of mice, researchers found that ETV1 was primarily expressed on ICC-MY and ICC-IM, and an ETV1 knockout mouse revealed absence of ICC-MY and ICC-IM [9]. These findings suggest that the development of ICC-MY and ICC-IM may be ETV1 dependent. The proposed mechanisms by which ETV1 exerts its effects include that ETV1 can positively regulate c-Kit expression through direct binding with the c-Kit enhancer regions, which then promotes c-Kit transcription [27]. ETV1 is a very unstable protein, and its stability depends on the active c-Kit signaling [9]. The decreased expression of ETV1 in the diabetic mice in our study suggested that the mSCF/Kit signaling may be inactive in these mice. This finding is in line with the reduced mSCF and c-Kit expression in diabetes. Both low-frequency and high-frequency EA could increase the ETV1 expression, and the present results also hinted that mSCF/Kit signaling may be recuperated to the activated state, which responds to the upregulated mSCF and c-Kit expression induced by the EA. A remarkable positive correlation was found between mSCF and ETV1, and the regression analysis further supports that mSCF is a potential determinant of ETV1 protein expression. Therefore, with EA stimulation, the mechanisms underlying the protection of the ICC in the antrum and corpus of diabetic mice may rely on the activation of mSCF/Kit-ETV1 signaling to facilitate c-Kit transcription and sustain the integrity of the ICC-MY and ICC-IM.

## 5. Conclusions

In conclusion, the decreased ICC networks in the antrum and corpus of diabetic mice could be well detected using c-Kit staining, and Ano1 is also a reliable and precise marker to monitor the changes of the ICC in diabetic mice. The increased ICC in the low- and high-frequency groups stained simultaneously by Ano1 and c-Kit further enhanced the effects of EA. The normal activation of mSCF/Kit-ETV1 signaling by low- and high-frequency EA may protect the networks of the ICC in diabetic mice at least in part. That may be a new view for us to understand the underlying mechanisms of EA and a deep investigation needs to be carried out in the future.
